# Continuous controlled-infusion of hypertonic saline solution in traumatic brain-injured patients: a 9-year retrospective study

**DOI:** 10.1186/cc10522

**Published:** 2011-10-28

**Authors:** Antoine Roquilly, Pierre Joachim Mahe, Dominique Demeure Dit Latte, Olivier Loutrel, Philippe Champin, Christelle Di Falco, Athanase Courbe, Kevin Buffenoir, Olivier Hamel, Corinne Lejus, Véronique Sebille, Karim Asehnoune

**Affiliations:** 1 Anesthesiology and Intensive Care Unit, Hôtel Dieu Nantes University Hospital, 1 place Alexis Ricordeau, Nantes, F-44093 France; 2 Neurosurgery Unit, Hôtel Dieu Nantes University Hospital, 1 place Alexis Ricordeau, Nantes, F-44093 France; 3 Cellule de Biostatistique, EA 4275, UFR de Pharmacie, Nantes University, A rue Gaston Veil, Nantes, F-44000 France

## Abstract

**Introduction:**

Description of a continuous hypertonic saline solution (HSS) infusion using a dose-adaptation of natremia in traumatic brain injured (TBI) patients with refractory intracranial hypertension (ICH).

**Methods:**

We performed a single-center retrospective study in a surgical intensive care unit of a tertiary hospital. Fifty consecutive TBI patients with refractory ICH treated with continuous HSS infusion adapted to a target of natremia. In brief, a physician set a target of natremia adapted to the evolution of intracranial pressure (ICP). Flow of NaCl 20% was *a priori *calculated according to natriuresis, and the current and target natremia that were assessed every 4 hours.

**Results:**

The HSS infusion was initiated for a duration of 7 (5 to 10) (8 ± 4) days. ICP decreased from 29 (26 to 34) (31 ± 9) mm Hg at H0 to 20 (15 to 26) (21 ± 8) mm Hg at H1 (*P *< 0.05). Cerebral perfusion pressure increased from 61 (50 to 70) (61 ± 13) mm Hg at H0 up to 67 (60 to 79) (69 ± 12) mm Hg at H1 (*P *< 0.05). No rebound of ICH was reported after stopping continuous HSS infusion. Natremia increased from 140 (138 to 143) (140 ± 4) at H0 up to 144 (141 to 148) (144 ± 4) mmol/L at H4 (*P *< 0.05). Plasma osmolarity increased from 275 (268 to 281) (279 ± 17) mmol/L at H0 up to 290 (284 to 307) (297 ± 17) mmol/L at H24 (*P *< 0.05). The main side effect observed was an increase in chloremia from 111 (107 to 119) (113 ± 8) mmol/L at H0 up to 121 (117 to 124) (121 ± 6) mmol/L at H24 (*P *< 0.05). Neither acute kidney injury nor pontine myelinolysis was recorded.

**Conclusions:**

Continuous HSS infusion adapted to close biologic monitoring enables long-lasting control of natremia in TBI patients along with a decreased ICP without any rebound on infusion discontinuation.

## Introduction

Refractory intracranial hypertension (ICH) is the most frequent cause of death after traumatic brain injury (TBI) [1]. In brain-injured patients, hyponatremia frequently develops, mainly caused by inappropriate antidiuretic hormone syndrome and cerebral salt-wasting syndrome [2,3]. Hyponatremia induces brain ischemia resulting from the swelling of perivascular astrocytic [4], and also increases the brain-contusion volume and intracranial pressure (ICP). The control of natremia is a major goal for prevention and treatment of ICH in an attempt to improve the neurologic recovery after brain injury.

The first-line treatment of ICH is osmotherapy [5]. HSS draws fluid from interstitial space, improves intracranial compliance, and decreases ICP, notably by counteracting the brain accumulation of extracellular osmolytes observed within blood-brain barrier dysfunction [6,7]. In this setting, a bolus of mannitol or of hypertonic saline solution (HSS) efficiently decrease the ICP [5,8]. Several reports suggested that a bolus infusion of HSS is more effective than mannitol for the treatment of elevated ICP, but mannitol is still the mainstay of hyperosmolar therapy [9,10]. A bolus of either mannitol or HSS encounters the same limits that are a time-limited effect as well as the risk of a rebound of ICH [5,8,9]. As the time-limited effect of a bolus of HSS is still an issue, several studies have evaluated the use of continuous HSS infusion after TBI. In adult patients, continuous HSS infusion has been tested prophylactically in the prevention of ICH, but no data are available in the setting of refractory ICH [11-14]. Continuous HSS infusion increased natremia and osmolarity, decreased the risk of ICH, and improved the cerebral perfusion pressure (CPP) in TBI patients without ICH [11-14]. To date, no clear conclusion can be drawn regarding potential side effects [15]. The HSS continuous infusion induced severe hypernatremia [12-14]. In this setting, dose adjustment of HSS is critical for preventing potential side effects of severe acute hypernatremia (osmotic demyelination syndrome or central pontine myelinolysis [16], renal failure [17], phlebitis [13]). Issues regarding side effects have not been addressed in the current literature. Moreover, data regarding the ending of the infusion are sparse, despite the risk of a rebound of ICP [11]. We present the report of our 9-years' experience with the use of an algorithm for dose adaptation of prolonged continuous HSS infusion in patients with refractory ICH. The aims of this descriptive study were therefore to describe a continuous infusion of HSS adapted to a target of natremia and to investigate its potential ability to decrease ICP without inducing severe hypernatremia and rebound in ICP in TBI patients with refractory ICH.

## Materials and methods

This retrospective descriptive study was performed from 1 January 2001 to 31 December 2009 in a surgical ICU of a tertiary hospital. No change in our current clinical practice and no randomization were performed. As it was an observational retrospective study, according to the French legislation (articles L.1121-1 paragraph 1 and R1121-2, Public Health Code), neither informed consent nor approval of the ethics committee was needed to use data for an epidemiologic study.

### Patients

Patients were identified from an electronic registry prospectively recorded. Inclusion criteria were as follows: (a) traumatic brain-injured patients defined as having a Glasgow Coma Score (GCS) **≤ **8 after initial care; (b) ICP monitored with an intraparenchymal probe; (c) treated with a continuous HSS infusion adapted to a target of natremia; and (d) in the purpose of refractory ICH. ICH was defined as an ICP > 20 mm Hg for more than 15 minutes [5,18]. Refractory ICH was considered when ICP remained > 20 mm Hg despite general care, control of capnia (< 5.8 kPa), as well as body temperature (< 38.0°C), and mannitol, and barbiturate injections [5]. Exclusion criteria were as follows: (a) continuous HSS infusion for < 8 hours, (b) or hyponatremia without intracranial hypertension.

### General care of severe head-trauma patients

All patients were sedated with a continuous intravenous infusion of fentanyl (2 to 5 μg.kg^-1^.hr^-1^) and midazolam (0.2 to 0.5 mg.kg^-1^.hr^-1^) and were mechanically ventilated. Apart from counterindications, sedated patients were kept in a semirecumbent position. Secondary brain injuries were prevented by keeping the body temperature between 36.0°C and 37.0°C, ensuring normoglycemia and normocapnia, and by avoiding hypoxemia. In severe TBI patients, natremia and blood gases were assessed at least twice a day, and expiratory end-tidal (Et) CO_2 _was continuously monitored. Patients were monitored with invasive arterial pressure, and mean arterial pressure was measured up to the brain for the calculation of the CPP. For patients with severe TBI and with an abnormal computed tomography (hematomas, contusions, swelling, herniation, or compressed basal cisterns), the ICP was monitored [5,19] with an intraparenchymal probe placed in the most affected side (Codman, Johnson and Johnson Company, Raynham, MA, USA.). CPP was maintained > 65 mm Hg with isotonic fluids (NaCl, 0.9%) and vasopressor (norepinephrine). Extraventricular drainage was used in case of hydrocephalus. Neuromuscular nondepolarizing agents were not used for ICH management. Mannitol (a bolus of 0.5 g/kg, repeatable once in case of poor ICP control, ICP > 20 mm Hg, after 30 minutes; maximal dose, 1 g/kg) was used to control episodes of ICH. When control of ICH was poor, midazolam infusion was continued, and barbiturate (sodium thiopental) was used (loading dose of 2 to 3 mg/kg) followed by a continuous infusion (starting dose of 2 to 3 mg/kg/h) adapted to the ICP evolution and, once per day, to a serum-level monitoring (thiopental blood level targets, 20 to 30 μg/ml) [20]. For each patient, continuous HSS infusion was started when the ICP remained > 20 mm Hg 30 minutes after the initiation of barbiturate infusion (refractory ICH). When the continuous osmotherapy failed (persistent refractory ICH), a decompressive craniectomy was discussed with the neurosurgical team, and HSS infusion was pursued.

### Adjustment of the target of natremia

**Figure 1:**
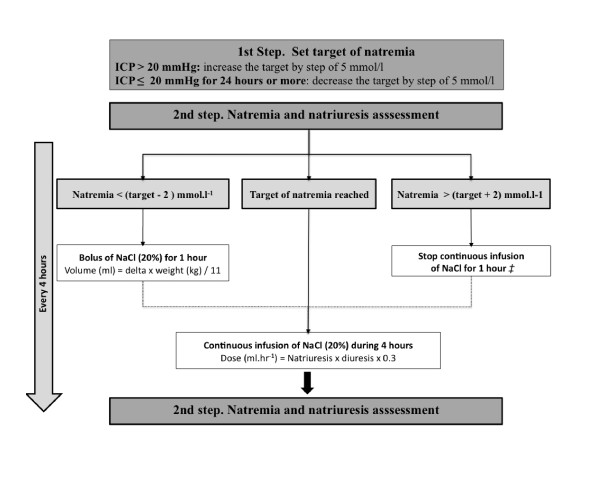
**Dose-adaptation of continuous hypertonic saline solution infusion**. The attending physician set the targets of natremia according to the intracranial pressure (ICP). The target could be modified by a step of 5 mmol/L from 145 to 155 mmol/L. Natremia and natriuresis were assessed every 4 hours. Target was considered achieved if -2 mmol/L < Delta < +2 mmol/L; otherwise, the flow of NaCl infusion was adapted. On infusion initiation or when natremia was below the target (left side), a 1-hour bolus was performed. When the target of natremia was reached (middle), the flow of a continuous infusion of HSS (NaCl 20%) was adapted to the urinary excretion of sodium, and the extraurinary sodium loss was neglected. If natremia was above the target (right side), the infusion of NaCl (20%) was discontinued for 1 hour. ‡Except in case of intracranial pressure > 20 mm Hg. Natriuresis, urinary sodium concentration (mmol/L); kaliuresis, urinary potassium concentration (mmol/L); dieresis, urinary output (ml/h); Delta, natremia - target).

The attending physician set a target of natremia (from 145 to 155 mmol/L) adapted to the evolution of ICP. When ICP was > 20 mm Hg, the target of natremia was increased by an increment of 5 mmol/L every 4 hours, and a bolus of HSS was infused (natremia below the new target, left side of Figure 1). The infusion of HSS was prolonged for as long as required to control the ICP. When the ICP was ≤ 20 mm Hg for at least 24 hours, the target of natremia was left unchanged until barbiturate infusion could be stopped. When barbiturate could be stopped (progressively) without increasing the ICP, the target of natremia was gradually decreased to 145 mmol/L (by decrements of 5 mmol/L) in an attempt to maintain the CPP and to prevent hyponatremia.

### Dose adaptation of a continuous HSS

To limit the risk of fluid overload observed with other saline solutions [11], we used a 20% chloride sodium solution infusion (adapted from [2,21,22]; see Figure 1 and Addition File 1 for an example). Dose-adaptation of HSS infusion was performed by nurses according to an algorithm (Figure 1). Biologic monitoring (blood and urinary electrolyte concentrations, osmolarity) was performed every 4 hours. For calculation, three situations were available:

1. On infusion initiation or when natremia was below the target, a bolus of chloride sodium was administered. The dose of sodium was calculated according to the natremia measured in the previous 12 hours (natremia below the target, Figure 1). Considering that a bolus of chloride sodium only fills the extracellular fluid compartment (one fourth of the body weight), the required volume of NaCl 20% was calculated as follows:

$$\text{Volume}\;\left(\text{NaCl 2}0\%\right)=\text{Delta}\times \text{weight}∕\text{11}.$$

The bolus was administered in 1 hour.

2. When the target of natremia was reached, the flow of continuous infusion of HSS (NaCl 20%) was adapted to the urinary excretion of sodium, and the extraurinary sodium loss was neglected. The flow of NaCl 20% was calculated as follows:

$$\text{Dose NaCl 2}0\%\left(\text{ml}∕\text{h}\right)=\text{Natriuresis}\times \text{Diuresis}\times 0.3$$

3. When the natremia was above the target, the infusion of NaCl (20%) was discontinued for 1 hour. Then the continuous infusion of NaCl was resumed for the remaining 3 hours.

A polyuria has been described with the intravenous infusion of HSS [11]. In an attempt to prevent severe dehydration, a compensation of the volume loss due to excessive diuresis should be performed. Replacement for volume loss was performed in 1 hour when diuresis exceed 120 ml/h (corresponding to basal hydration excluding HSS infusion) with a solution composed as follows: 1,000 ml Glucose (2.5%) + NaCl (natriuresis/17) grams + KCl (kaliuresis/13) g.

### Definitions

Local normal ranges for natremia were 137 to 145 mmol/L. Acute kidney injury was defined by a 200% increase in serum creatinine concentration as compared with a previous assessment of renal function [23]. Severe central pontine myelinolysis was considered if clinical symptoms (prolonged alteration of consciousness, quadriplegia, and dysarthria) were associated with the appearance of a central pons lesion on magnetic resonance imaging.

### Data collection

The following data were recorded in the electronic medical file prospectively completed: age, sex, GCS, ICH management, continuous infusion of HSS (duration, target natremia, biologic monitoring, blood osmolarity measured with an osmometer, quantity of infused sodium), and evolution of ICP and CPP during the infusion. Serum creatinine concentration, coagulation tests, and central pontine myelinolysis were also recorded, as well as the Glasgow Outcome Scale (GOS) and death at 1 year.

### Statistical analysis

To account for the correlation between measurements from the same individual, repeated measures analysis of variance (ANOVA) using linear mixed models, allowing random effects with restricted maximum-likelihood estimation, was used to examine changes in variables over time. Time effect was included in the models along with baseline measurements. Several covariance structures among the repeated measurements (autoregressive, unstructured, Toeplitz, and so on) were compared by using Akaike's Information Criterion [24]. Residual analysis was used to evaluate the validity of the models assumptions, including normality and homoscedasticity. Mixed models *post hoc *tests based on estimated marginal means were performed for comparing the levels of the studied variables at different times. Skewed variables were log-transformed, and statistical analyses were performed with SAS 9.1 statistical software (SAS Institute, Cary, NC, USA). Continuous variables were expressed as median (percentiles 25 to 75; mean ± SD). The *P *values for each variable tested are presented in Additional file 1, Table S1. *P *values < 0.05 were considered to be statistically significant.

## Results

### Population

**Figure 2:**
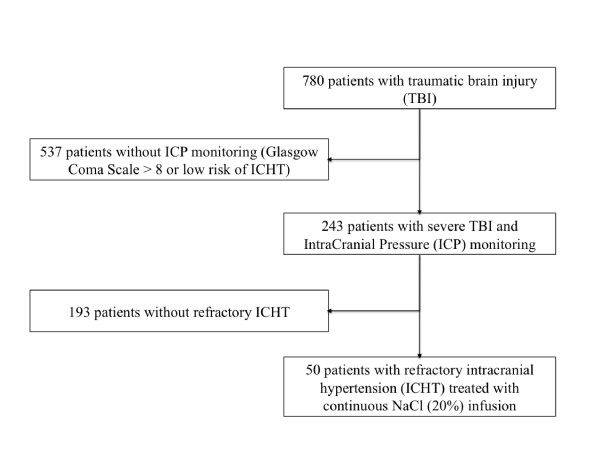
**Flow chart**. ICHT, intracranial hypertension; ICP, intracranial pressure; TBI, traumatic brain injury.

During the 9-year study period, 780 patients with TBI were admitted into the ICU. Among the 243 (31.2%) patients with a severe TBI monitored with an ICP, 50 (20.6%) patients developed a refractory ICH and were treated with a continuous HSS infusion (Figure 2). Patients were aged 40 (range, 25 to 45 years) (36 ± 13) years, and 46 (92%) were men. The GCS on the scene was 6 (5 to 8; 6 ± 2), and the first measured ICP was 28 (25 to 34; 31 ± 9) mm Hg (Table 1). Traumatic lesions observed on the initial cerebral tomodensitometry were 24 (48%) subarachnoid haemorrhage, 19 (38%) epidural haemorrhage, 40 (80%) cerebral contusions, and no patient had hydrocephalus (multiple lesions could be observed in the same patient). Of 50 patients, 29 (58%) required neurosurgery for hematoma evacuation. Barbiturate infusion was started on day 2 (1 to 3; 3 ± 2) for a duration of 5 (4 to 8; 6 ± 3) days. Over the period of infusion, the dose of barbiturate was 2.7 (1.4 to 5.5; 3.1 ± 2.5) mg/kg/h. The length of stay in the ICU was 25 (9 to 36; 27 ± 19) days. Of 50 patients, three (6%) died in the ICU of intractable ICH, and 10 (20%) of care withdrawal. The GCS at discharge from the ICU (for surviving patients) was 14 (8 to 15; 13 ± 7), and the GOS evaluated at 1 year was 4 (1 to 5; 3 ± 2; Table 1).

**Table 1:** Population description

Characteristics	TBI patients with refractory intracranial pressure (*N *= 50)	
	*n *(%) or median (IQR)	Mean ± SD
Age (years)	40 (25-45)	**36 ± 13**
Male *n (%)*	46 (92)	
ISS *n (%)*	29 (25-34)	**29 ± 6**
SAPS 2	45 (40-47)	**45 ± 9**
Glasgow Coma Scale on scene	6 (5-8)	**6 ± 2**
Diagnosis *n *(%)		
Subarachnoid hemorrhage	24 (48)	
Cranial extradural hematoma	19 (38)	
Cerebral contusion	40 (80)	
Initial intracranial pressure (mm Hg)	28 (25-34)	**31 ± 9**
Initial values before starting HSS infusion		
Intracranial pressure (mm Hg)	29 (26-34)	**31 ± 9**
Cerebral perfusion pressure (mm Hg)	61 (50-70)	**61 ± 13**
Natremia (mmol/L)	140 (138-143)	**140 ± 4**
Plasma osmolarity (mmol/L)	275 (268-281)	**279 ± 17**
Norepinephrine before starting HSS infusion *n *(%)	49 (98)	
ICU length of stay (days)	25 (9-36)	**27 ± 19**
Glasgow Coma Scale at ICU discharge for survival patients	14 (8-15)	**13 ± 7**
Death in ICU *n *(%)	13 (26)	**/**
Glasgow Outcome Scale at 1 year		
1 *n *(%)	17	
2 *n *(%)	4	
3 *n *(%)	4	**/**
4 *n *(%)	12	
5 *n *(%)	13	

ISS, Injury Severity Score; SAPS, simplified acute physiology score; Cerebral perfusion pressure, (Mean arterial pressure - Intracranial pressure); ICU, intensive care unit; HSS, hypertonic saline solution; Glasgow Outcome Scale ranges: 1, dead; 2, vegetative state (meaning the patient is unresponsive, but alive); 3. severely disabled (conscious, but the patient requires others for daily support because of disability); 4, moderately disabled (the patient is independent but disabled); 5, good recovery (the patient has resumed most normal activities but may have minor residual problems).

The continuous HSS infusion was started on day 2 (1 to 4; 3 ± 2) for a duration of 7 (5 to 10; 8 ± 4) days. After the first 96 hours, the number of patients still receiving continuous HSS decreased considerably (discontinuation of HSS infusion or death); see Additional File 1, Figure S1. In an attempt to preserve the statistical power of the study, we provide the results for the first 96 hours of infusion (H0 to H96).

### Evolution of ICP and CPP

**Figure 3:**
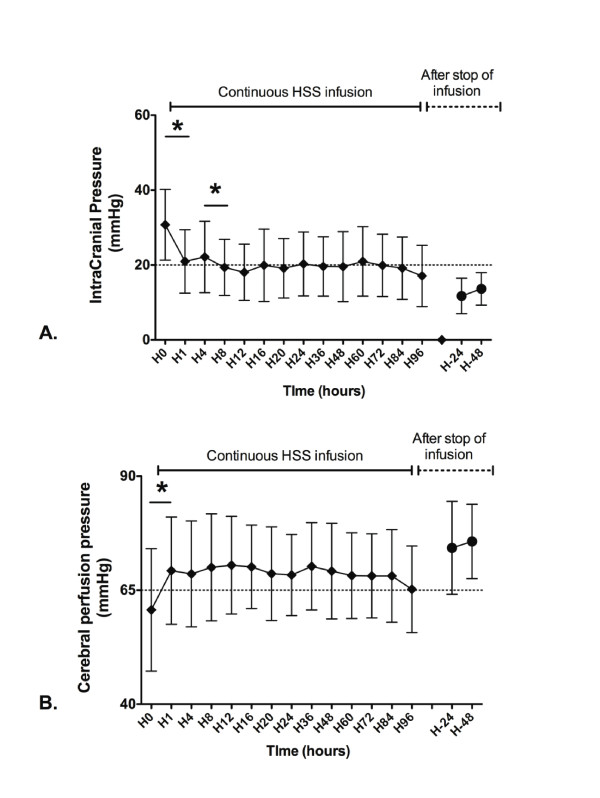
**Time evolution of (a) intracranial pressure and (b) cerebral perfusion pressure during and after the continuous HSS infusion**. Cerebral perfusion pressure was calculated as follows: Mean arterial pressure - Intracranial pressure. Results were provided for the first 96 hours of HSS infusion (from H0 to H96; white boxes) and 2 days after the stop of infusion (from day 1 to day 2; gray boxes). *P < 0.05.

ICP significantly diminished from 29 (26 to 34; 31 ± 9) mm Hg at H0 down to 20 (15 to 26; 21 ± 8) mm Hg at H1 (*P *< 0.05 versus H0), and from 22 (15 to 28; 22 ± 9) mm Hg at H4 to 20 (15 to 24; 19 ± 7) mm Hg at H8 (*P *< 0.05 versus H4). Afterward, ICP was stable until H96 (Figure 3a). When the HSS infusion was stopped, the ICP remained unchanged from 11 (8 to 14) (12 ± 5) mm Hg at H-24, to 13 (10 to 17) (14 ± 4) mm Hg at H-48 (non significant, NS; Figure 3a). Cerebral perfusion pressure (CPP) increased from 61 (50 to 70) (61 ± 13) mm Hg at H0 up to 67 (60 to 79) (69 ± 12) mm Hg at H1 (*P *< 0.05). CPP was stable until H96 (Figure 3b). When stopping the HSS infusion, CPP remained unchanged from 72 (64 to 86) (74 ± 10) mm Hg at H-24, to 74 (68 to 83) (76 ± 8) mm Hg at H-48 (NS; Figure 3b). A decompressive craniectomy was performed for five patients (10%) for a persistent ICH (one patient died in the ICU, and GOS at 1 year was equal to 3 for one, to 4 for two, and to 5 for one patient). Capnia, body temperature, vasoactive drugs were not significantly modified during the infusion (data not shown).

### Evolution of natremia and osmolarity

**Figure 4:**
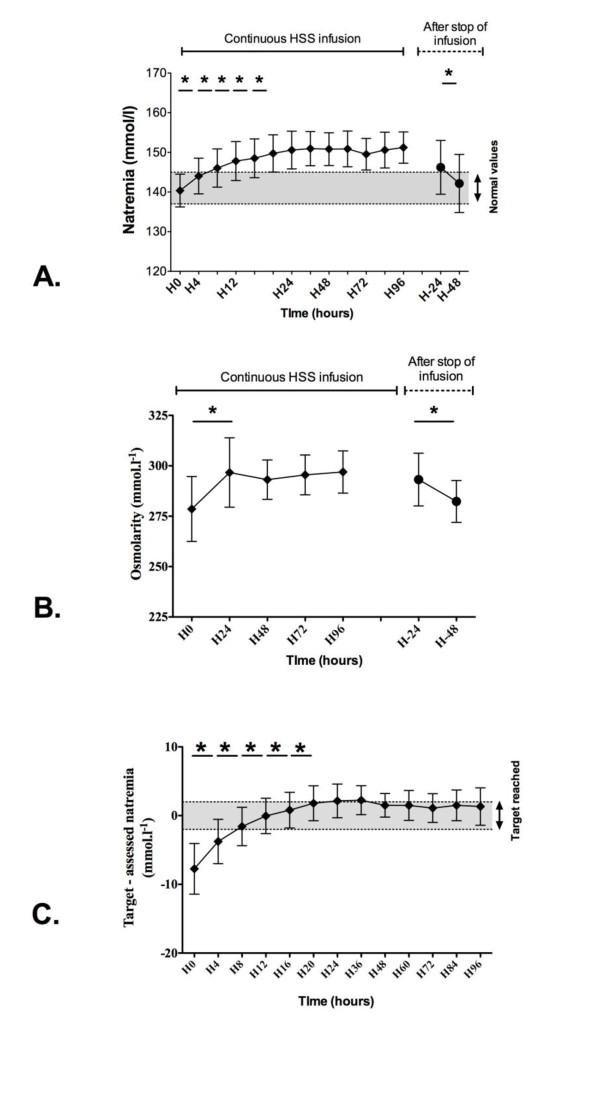
**Time evolution of (a) natremia, (b) blood osmolarity, and (c) delta of natremia during and after the continuous HSS infusion**. **(a) **Natremia was assessed every 4 hours. **(b) **Osmolarity was assessed once per day. Results are given for the first 96 hours of HSS infusion (white boxes) and 2 days after the stop of infusion (gray boxes). **(c) **Delta between assessed natremia and target of natremia was calculated every 4 hours. Target was considered as achieved if -2 mmol/L < Delta ≤ 2 mmol/L. Results are given for the first 96 hours of HSS infusion (white boxes). *P < 0.05.

Natremia increased from 140 (138 to 143) (140 ± 4) at H0 to 144 (141 to 148) (144 ± 4) mmol/L at H4 (*P *< 0.05; Figure 4a). Afterward, natremia continuously increased until the 20th hour of HSS infusion, and remained stable until the 96th hour (Figure 4a). When we stopped the HSS infusion, natremia decreased from 146 (142 to 151) (146 ± 7) mmol/L at H-24, to 141 (139 to 148) (142 ± 7) mmol/L at H-48 (*P *< 0.05, Figure 4a). Osmolarity increased from 275 (268 to 281) (279 ± 16) mmol/L at H0 up to 290 (284 to 307) (297 ± 17) mmol/L at H24 (*P *< 0.05), and remained stable until H96 (Figure 4b). Osmolarity decreased from 292 (283 to 303) (293 ± 13) mmol/L at H-24 after the infusion ended, to 281 (276 to 287) (282 ± 10) mmol/L at H-48 (*P *< 0.05; Figure 4b). The target of natremia was reached as soon as H8, and natremia remained in the target range until H96 (Figure 4c).

### Time evolution of the quantity of infused-sodium

The amount of sodium administered was not significantly different during the study period: 32 (17 to 46) (36 ± 23) g in the first 24 hours, and 25 (11 to 45) (32 ± 28) g from H72 to H96 (NS). After we stopped the HSS infusion, a bolus of 7 (5 to 11) (7 ± 4) g of sodium (flow, 1 g/hour) was administered at H-24, and a bolus of 1 (0 to 4) g (2 ± 3) was administered at H-24 (*P *< 0.05). Natriuresis increased from 25 (20 to 40) g (30 ± 18) in the first 24 hours up to 31 (24 to 45) (38 ± 25) g from H24 to H48 (*P *< 0.05) and remained unchanged afterward (NS). Natriuresis decreased from 18 (13 to 20) (17 ± 5) g/day at H-24 after the infusion ended, down to 12 (9 to 16) (12 ± 4) g/day at H-48 (*P *< 0.05).

### Side effects

**Figure 5:**
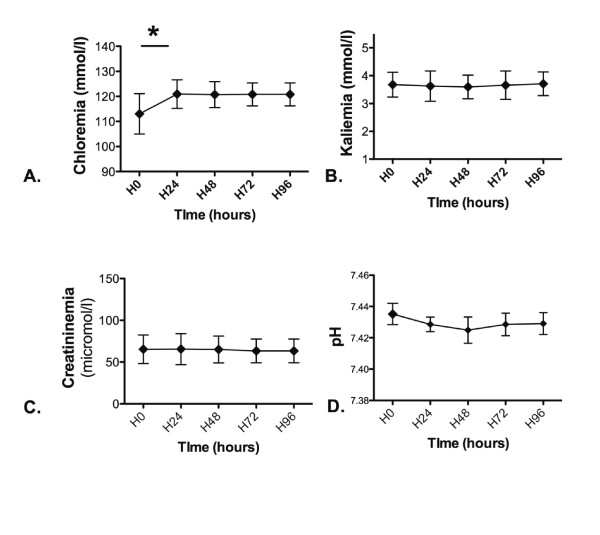
**Time evolution of (a) chloremia, (b) kaliemia, (c) creatininemia, and (d) pH during the continuous HSS infusion**. Results of **(a) **chloremia, **(b) **kaliemia, (c) creatininemia, and **(d) **pH were collected every 24 hours. Results were provided for the first 96 hours of HSS infusion (unchanged for longer duration). *P < 0.05.

Chloremia increased from 111 (107 to 119) (113 ± 8) mmol/L at H0 to 121 (117 to 124) (121 ± 5) mmol/L at H96 (*P *< 0.001; Figure 5a). Kaliemia, creatininemia, and pH remained unchanged within the study intervention (NS; Figure 5b-d). No pulmonary edema was recorded. Severe-form centropontine myelinolysis was neither clinically suspected nor confirmed by magnetic resonance imaging.

## Discussion

The use of a continuous HSS controlled infusion adapted to a target of natremia for treating post-traumatic refractory ICH allowed us to decrease ICP and to rapidly improve CPP without any severe acute hypernatremia.

Hyponatremia and hypo-osmolarity are frequent in TBI patients and induce an increase in ICP as well as in the volume of intracranial lesions [4]. Hyperosmolar therapy with either mannitol or HSS reduces ICP [8-11,25] and is recommended for the treatment of ICH [5]. Boli of hyperosmolar solutions decrease ICP for less than 6 hours and expose patients to a rebound of ICH [8,9,26]. After a bolus of osmotherapy, the increase in cerebrospinal fluid osmolarity may participate in the risk of rebound of ICP [27]. During osmotherapy, a decrease in extracellular volume contributes to the fast control of ICP [28], but an accumulation of organic osmolytes (slow adaptation) [29] may limit the decrease in brain volume and may expose the patient to a rebound of ICP. In this setting, continuous osmotherapy was proposed in the 1990s but is still not recommended. Side effects reported in case of improper use of mannitol (dehydration, renal failure, hypotension) may have limited its use as a continuous therapy, and to date, only HSS infusion has been tested for continuous osmotherapy. Moreover, HSS has a higher reflection coefficient than mannitol that may decrease the risk for rebound.

In a randomized study in TBI patients, continuous infusion of HSS for 5 days increased both natremia and osmolarity but did not decrease ICP, as compared with hypotonic infusion [30]. In this study, the HSS-treated group had a significantly worse neurologic status with higher ICP on inclusion than did the hypotonic-treated group. Interestingly, no side effects were reported. In two other studies [12,13], a continuous infusion of 3% saline in TBI patients without *a priori *dose adaptation decreased ICP but did not alter outcomes. In patients with severe cerebrovascular disease and at high risk for ICH, the preventive continuous infusion of HSS (3%) may reduce the frequency of ICP crises and the mortality rate [14]. In patients with posttraumatic/operative edema and treated with a continuous 3% HSS infusion to a target natremia of 145 to 155 mmol/L [11], an inverse relation between natremia and reduction of both ICP and brain edema was found. Finally, continuous infusion of HSS is an attractive treatment in an attempt to achieve long-lasting control of ICP (for a review see [15]), but few data were available in patients with refractory ICH. Consistent with a previous report, the current protocol of continuous infusion of HSS (NaCl 20%) was associated with a rapid and prolonged decrease of ICP values in patients with ICH refractory to barbiturate.

In this study, a 20% saline infusion was used to decrease the risk of fluid overload, and no pulmonary edema or acute kidney injury was recorded. However, because of the retrospective design of our study, the potential side effects of HSS were recorded from the ICU report. The risk of uncontrolled metabolic disorder associated with HSS infusion remains problematic, poorly described, and has probably limited the widespread use of HSS [15]. In two studies [12,13], continuous infusion of 3% saline in TBI patients without *a priori *dose adaptation decreased ICP but induced severe hypernatremia that reached up to 180 mmol/L, and concerns regarding neurologic complications and kidney failure have been raised. In the study by Qureshi *et al. *[11], risks of fluid overload (pulmonary edema) and of metabolic complications (kidney failure, polyuria) with a continuous infusion of saline, 3%, have resulted in poor outcomes. According to previous reports [11-14,30], in no patient did a severe form of pontine myelinolysis develop. The cases of pontine myelinolysis reported linked to a fast increase in natremia were observed in patients with chronic hyponatremia [16] but not with infusion of HSS for posttraumatic ICH. In a description of HSS infusion in TBI children, no osmotic demyelination syndrome was seen on magnetic resonance imaging, even for natremia reaching 171 mmol/L [12].

The main side effect reported in the present report was hyperchloremia. Excessive chloride infusion is a major factor with hyperchloremic acidosis. Thus, it is likely that hyperchloremic acidosis developed in TBI patients treated with continuous HSS infusion. Hyperchloremic acidosis increases the symptoms of postoperative ileus and induces biologic hemostasis perturbation [31]. To date, these complications are not evidence based in the ICU setting.

Finally, a dose adaptation of HSS infusion with rigorous biologic monitoring may explain the lack of an uncontrolled metabolic disorder, except for a probable hyperchloremic acidosis, in our results.

How to stop the infusion is of major importance to prevent a rebound of ICH, and few data are available. In the study by Qureshi *et al. *[11], half of the study population experienced a relapse of ICH at the end of the infusion. Interestingly, even with a likely residual barbiturate blood level, we did not report any rebound in ICP after stopping the infusion. In the current algorithm, the slow tapering of continuous HSS treatment could have restored a normal brain osmolality without inducing a cerebral edema, as the dissipation of accumulated electrolytes and organic osmolytes takes place along with water repletion [6,28,32]. Finally, a slow reduction of the flow may be recommended in case of continuous HSS infusion.

Several therapeutics have been tested for decreasing ICP in case of refractory ICH. Despite an improvement of the neurologic recovery in patients with moderate to severe TBI [33], moderate hypothermia may expose patients to intracranial bleeding as well as secondary infections [34] and is not currently recommended in patients with refractory ICH [5]. Surgical craniotomy is another option, but the optimal timing to perform this procedure remains controversial, and its efficiency to enhance neurologic recovery is still debated [18,35]. Regarding the absence of severe side effects with the current protocol, continuous controlled infusion of HSS may thus be an interesting alternative for the treatment of refractory ICH.

Our study encountered limitations. First, the study had a retrospective design, and a randomized trial is necessary to confirm these results. Second, the external validity of this single-center study may be limited. However, our findings may be relevant to the vast majority of level I trauma centers routinely practicing TBI management. We did not report any significant adverse events related to the use of continuous HSS infusion, but this study is not powered to confirm its safety. Finally, a comparison with an untreated group would have strengthened our conclusions, and a larger study with a prospective randomized design is required.

## Conclusions

We describe for the first time a well-tolerated and reproducible adaptation of continuous HSS infusion for refractory ICH that relies on a closed biologic control of natremia. An increased CPP together with a decrease in ICP was observed during continuous HSS infusion without any development of severe hypernatremia or rebound of ICP. Continuous infusion of HSS with a dose adaptation to a target natremia could therefore be an attractive alternative for patients treated with barbiturates for refractory ICH. Prospective studies are required to confirm the effects and safety of the current dose adaptation of HSS infusion.

## Key messages

▪ Continuous hypertonic saline infusion may decrease intracranial pressure in traumatic brain-injured patients with refractory intracranial hypertension

▪ The current dose adaptation of hypertonic saline infusion, based on a target of natremia, is reliable and well tolerated

▪ Close biologic monitoring within continuous hypertonic saline infusion prevents severe hypernatremia

▪ No rebound of intracranial pressure was observed after the infusion ended

▪ Continuous infusion of hypertonic saline infusion could be an attractive alternative to treating patients with refractory intracranial hypertension

## Abbreviations

CPP: Cerebral perfusion pressure; GCS: Glasgow Coma Scale; GOS: Glasgow Outcome Scale; HSS: hypertonic saline solution; ICH: intracranial hypertension; ICP: intracranial pressure; ICU: intensive care unit; TBI: traumatic brain injury.

## Competing interests

The authors declare that they have no competing interests.

## Authors' contributions

AR, PJM, DD, OL, PC, CL, and KA designed the study. AR, PJM, DD, CDF, AC, OL, and PC collected the clinical information. VS analyzed the raw data, performed statistical analysis, and drafted and contributed to the writing of the article. AR, PJM, DD, OL, PC, CDL, AC, KB, OH, and KA included patients, and drafted and contributed to the writing of the article. CL participated in the interpretation of all data, revising the manuscript critically for important intellectual content. All the authors contributed to the final approval of the manuscript.

## Supplementary Material

Additional file 1 **Table S1**. Linear mixed-models analyses of the evolution of intracranial pressure, cerebral perfusion pressure, delta natremia, osmolarity, kaliemia, chloremia, creatininemia, and natriuresis with time. **Figure S1**. Kaplan-Meier curve for the number of patients treated with continuous HSS. Click here for file
