# Supplementary material for: Continuous controlled-infusion of hypertonic saline solution in traumatic brain-injured patients: a 9-year retrospective study
Source: Crit Care. 2011 Oct 28;15(5):R260. doi: 10.1186/cc10522 (PMC3334811; doi:10.1186/cc10522)
Supplement: Additional file 1 — Table S1. Linear mixed-models analyses of the evolution of intracranial pressure, cerebral perfusion pressure, delta natremia, osmolarity, kaliemia, chloremia, creatininemia, and natriuresis with time. Figure S1. Kaplan-Meier curve for the number of patients treated with continuous HSS. [file cc10522-S1.DOC]

**Supplemental File 1**

**Rationale of the protocol**

Three caclulations are usefull for the dose-adaptation of HSS infusion :

A) Natremia is below the target and a 1-hour bolus is required in an attempt to increase ntatremia.

B) Natremia reaches the target and only the compensation of urinary sodium loss is required.

C) Compensation of polyuria

1. **Dose of “1- hour bolus”**

Adrogue et al. *(Intens Care Med 1997, and N Engl J Med 2000)* have proposed a formula to treat hyponatremia:

**Sodium requirement = (desired [Na+] – current [Na+]) x Total body water**

The estimated total body water (in liters) is calculated as a fraction of body weight. The fraction is 0.6 and 0.5 in non elderly men and women, respectively; and 0.5 and 0.45 in elderly men and women, respectively (Oh et al. Nephron 1995). The formula could be simplified as follows:

**Sodium requirement = Delta Natremia x Weight x 0.6**

Hypernatremia due to hypertonic sodium gain results in *(Adrogue et al. N Engl J Med 2000):*

- Extracellular: increase of both NaCl pool and extracellular fluid volume
- Intracellular: NaCl pool remains constant and intracellular fluid volume decreases.

We have thus considered that the amount of NaCl infused during HSS infusion would only fill the extracellular sector, approximatively 40% of total body water in healthy patients *(Oh et al. Fluid, electrolyte, and acid-base disorders. 2nd ed. New York: Churchill Livingstone, 1995:1-28)* and of 50% in ICU patients.

**Sodium requirement = Delta Natremia x Weight x 0.3**

We have standardised the use of NaCl 20% (3.4 mmol.ml^-1^). The formula could thus be approached by:

**Volume (NaCl 20%) = (Delta x weight) x 0.3 / 3.4 = Delta x weight / 11**

1. **Dose of “Continuous HSS infusion”**

On steady state (Natremia reaches the target), only the compensation for sodium lost is required.

Given the flow of sodium in the urines during HSS infusion, we have estimated that the extra-urinary sodium loss can be negligee.

**Dose NaCL 20% (mL/hr) = Natriuresis x Diuresis x 0.3**

1. **Compensation of polyuria**

*1) Replacement for volume loss due to diuresis: threshold:*

A polyuria has been described when using intravenous infusion of HSS (*Qureshi et al. Crit Care Med 1998*). In an attempt to prevent severe dehydration, a compensation of the volume loss due to excessive diuresis should be performed. The definition of polyuria is controversial issue in TBI patients. In our protocol, the basal hydration in TBI patients, excluding HSS infusion, reaches 40 ml.kg^1^.day^-1^ composed as follows:

- The flow of enteral nutrition (1 kCal.ml^-1^) reaches 35 ml.ml^-1^.day^-1^ in order to reach the recommended dose of 35 ml.kCal^-1^.day^-1^ *(Martindale et al. Crit Care Med 2009).*
- The flow of intravenous drugs frequently reaches (5 ml.kg^-1^.day^-1^, unpublished data).

Finally, a threshold of 120 ml.hr^-1^ representing 40 ml.kg^1^.day^-1^ approximatively for an adult of 70 kg was determined in order to compensate only urinate flow exceeding basal hydratation.

*2) Composition of the solution used for diuresis compensation*

To ease the understanding of the calculations, the amounts of NaCl and of KCl are now expressed in gram and no more in ml. The calculations aimed to convert mmol.l^-1^ (unit of natriuresis used by our laboratory) to grams in order to **facilitate** the solution preparation by nurses. Molar mass of NaCl is approximatively 17 gram.mol^-1^ and of 13 gram.mol^-1^ for KCl.

**Replacement for volume loss was performed when diuresis exceed 120 ml.hr^-1^ (corresponding to basal hydration excluding HSS infusion) with a solution composed as follow : 1000 ml Glucose (2.5%) + NaCl (natriuresis/17) grams + KCl (Kaliuresis /13) grams.**

**Exemple**

A man of 70 kg, with a natremia of 140 mmol/L, is developping a refractory intracranial hypertension. The attending physician set a target of natremia of 145 mmol/L (Delta = 5). The last 4-hours diuresis is 0.05 l. Immediately before the bolus, an urinary sample is harvested and the natriuesis is measured ( = 200 mmol/L)

**A) 1- hour bolus**

Volume (NaCL 20%) = Delta x weight / 11 = 5 : 70 / 11 = 31.8 ml (6.4 grams).

**B) Continuous infusion of NaCL 20%**

Volume (NaCl 20%) = Natriuresis x Diuresis x 0.3 = 200 x 0.05 x 0.3 = 3 ml / hr (0.6 grams/hr)

Additional Table S 1. Linear mixed models analyses of the evolution of ICP, CPP, delta Natremia, osmolarity, kaliemia, chloremia, creatininemia and natriuresis with time.

| Variables | Effects | Estimate | SE | P-values |
| --- | --- | --- | --- | --- |
| Log (ICP) | Baseline | 0.595 | 0.181 | 0.002 |
|  | Time | -0.002 | 0.001 | 0.001 |
| CPP | Baseline | 0.264 | 0.049 | <0.001 |
|  | Time | -0.040 | 0.020 | 0.045 |
| Delta Na_basal_ | Baseline | 0.092 | 0.056 | 0.108 |
|  | Time  Time^2^ | 0.446  -0.008 | 0.035  0.001 | <0.001  <0.001 |
| Log (osmolarity) | Baseline | 0.510 | 0.040 | <0.001 |
|  | Time | 0.0001 | 0.0001 | 0.120 |
| Kaliemia | Baseline | -0.139 | 0.148 | 0.352 |
|  | Time | 0.014 | 0.040 | 0.732 |
| Chloremia | Baseline | 0.388 | 0.062 | <0.001 |
|  | Time | 0.027 | 0.357 | 0.940 |
| Creatininemia | Baseline | 0.777 | 0.063 | <0.001 |
|  | Time | -0.848 | 0.943 | 0.371 |
| Natriuresis | Baseline | 0.861 | 0.153 | <0.001 |
|  | Time | 0.607 | 1.584 | 0.703 |

ICP: Intra Cranial pressure; CPP: Cerebral Perfusion Pressure; the “Delta Na_basal_” was : basal natremia (before protocol institution)- T natremia.

**Additional Figure S1: Kaplan-Meier curve for number of patients treated with continuous HSS.**

**
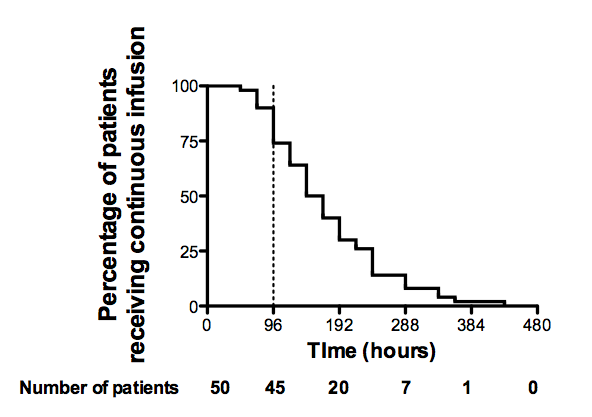
**
